# Phenotypic heterogeneity of latent autoimmune diabetes in adults identified by body composition analysis

**DOI:** 10.1186/1758-5996-6-128

**Published:** 2014-11-25

**Authors:** Giovanni Mario Pes, Alessandro Palmerio Delitala, Giuseppe Delitala, Alessandra Errigo, Salvatore Costantino, Giuseppe Fanciulli

**Affiliations:** Department of Clinical and Experimental Medicine, University of Sassari, Viale San Pietro 8, I-07100 Sassari, Italy

**Keywords:** Latent autoimmune diabetes in adults, Body composition, Insulin dependence

## Abstract

**Background:**

In patients with Latent Autoimmune Diabetes in Adults (LADA) a lower body mass index was reported compared with classical type 2 diabetes (T2D), and was found to be associated with a faster progression to insulin-dependence. In this study we determined the body composition in a cohort of LADA patients from Sardinia, Italy, and compared it with age– and gender–matched patients diagnosed as having adult-onset type 1 diabetes (T1D) and non-autoimmune T2D.

**Methods:**

In 210 LADA patients, 210 T2D patients and 30 adult-onset T1D patients of Sardinian origin we assessed total and segmental body composition (weight-adjusted percent fat mass and lean mass) by using Dual Energy X-rays Absorptiometry (DXA).

**Results:**

In the whole cohort of LADA patients total fat mass was significantly smaller compared with T2D patients (p < 0.0001), while no difference was found between LADA and T1D patients. In LADA men fat depletion involved all body segments, while in LADA women it was observed only in the truncal segment (p < 0.0001), as in the upper and lower regions fat deposits were larger compared to T2D (p < 0.0001). However, LADA women showed a significantly elevated truncal fat compared to T1D women (p < 0.004), whereas no difference was detected in the extremities.

**Conclusions:**

Body composition in LADA patients shows substantial difference, in a gender-dependent way, compared to classic T2D. In women fat deposits tend to accumulate in peripheral regions rather than centrally, whereas in men the distribution is more homogeneous. In addition, central fat depletion in LADA women appears to be a significant predictor of faster progression to insulin dependence. Thus, routine assessment of body composition may help the physician identify LADA patients who require early insulin treatment in order to delay beta-cell exhaustion, as well those with increased CV risk due to excess truncal adiposity.

## Background

Approximately 5-10% of patients initially diagnosed as having type 2 diabetes (T2D) mellitus show markers of autoimmunity against pancreatic beta-cells, such as serum antibodies to glutamic acid decarboxylase (GADAb), tyrosine phosphatase–like protein (I–A2Ab), and islet cell cytoplasmic antigen (ICA) [1]. This form of diabetes, referred to as “latent autoimmune diabetes in adults” (LADA) is often considered a slow-developing form of type 1 diabetes (T1D) [2, 3]. However, many clinical features of LADA, including onset in adult life, absence of ketoacidosis and slower progression towards insulin dependence, resemble more closely T2D than T1D. Similarities with T2D were reported also as regards anthropometric and metabolic data, although literature data are not entirely consistent. Family studies such as the Botnia Study in western Finland [4], which investigated a large cohort of GADAb-positive diabetic patients, reported that average body mass index (BMI), waist-to-hip ratio, and frequency of hypertension were lower – and the level of HDL cholesterol higher – compared with antibody-negative T2D patients. In contrast, the Diabetes Outcome Progression Trial (ADOPT) study did not find any significant difference between LADA and T2D in regard to BMI, blood pressure, glycated hemoglobin and lipid profile [5]. However, in this study the average waist circumference and the frequency of metabolic syndrome were significantly lower in LADA than in T2D patients, a finding also reported in a survey of T2D patients from five European countries, including Italy [6]. Besides these phenotypic differences between LADA and T2D, a number of studies revealed a considerable clinical heterogeneity even within LADA itself, often reflecting underlying immunological diversity (e.g. presence of single or multiple antibodies, variability in GADAb titers). Lohmann and coworkers reported that diabetic patients with elevated GADAb titers, or presence of multiple antibodies to beta-cells (defined as “LADA–1 patients”), have lower mean BMI values and a lower prevalence of long–term complications compared with patients with a single antibody positivity and/or low titers of circulating antibodies (defined as “LADA–2 patients”) [7]. In line with these findings, we recently reported, in a LADA cohort from Sardinia, a lower mean value and larger variability of BMI in comparison with T2D patients of the same ethnic origin, and we provided evidence that a BMI value below 28 kg/m^2^ is a strong predictor of progression towards insulin-dependence within 4 years [8]. This finding raises the general question as to whether a particular distribution of body fat in this form of diabetes can influence the clinical outcome. Only relatively few investigations have described the distribution of BMI in LADA [4–8] and no systematic studies were focused so far on body composition (BC) in this form of diabetes, using appropriate and validated instrumental procedures such as Dual energy X-ray absorptiometry (DXA) [9]. Moreover, since central obesity is a well recognized risk factor for cardiovascular (CV) disease, the assessment of the relative amount of fat and its distribution into the body compartments might help the physician identify sub–groups of patients with increased CV risk, thereby improving the prevention of long–term complications in this form of diabetes. The aim of the present study was to determine the BC, in a previously recruited cohort of LADA patients from Sardinia, by using DXA scan which is currently considered the clinical gold standard for BC assessment, and to evaluate the fat and lean mass distribution in comparison with two control groups, i.e. antibody-negative T2D patients and adult-onset (> age 30) T1D patients. Finally, we evaluated the impact of BC on the progression towards beta-cell failure.

## Methods

The characteristics of LADA patients have been described in our previous study [8]. Of the original cohort of 251 patients, 210 (118 women and 92 men, median age 54 years, range 35-65 years) agreed to undergo a full BC assessment and their clinical characteristics were representative of the original cohort. All LADA patients did not receive insulin therapy over the next 6 months after diagnosis. The duration of diabetes lasted from no less than 8 months to no more than 5 years, and none of these patients at onset presented with ketoacidosis and/or significant weight loss. Two control groups were also recruited: (i) 210 age – and gender–matched T2D patients who were demonstrably GADAb–negative; (ii) 30 T1D patients whose diabetes had developed after the age of 30 but had displayed ketoacidosis and/or had received insulin treatment in the 6 month after diagnosis. In this group of patients the sudden onset and the immediate requirement of insulin replacement therapy were considered sufficient to warrant the diagnosis of T1D, therefore GADAb antibodies were not determined.

All study participants were of Sardinian origin for at least two generations and none of them showed signs of severe liver or kidney disease. The study was approved by the local Ethics Committee (University of Sassari, Sardinia, Italy), and all participants provided a signed informed consent to participate in the study.

In all study participants body height (m) was measured using a stadiometer and body weight with an electronic balance with accuracy up to 0.1 kg. BMI was calculated as weight/height^2^ (kg/m^2^). Waist circumference (cm) was measured at the iliac crests in the upright position.

Whole-body and segmental DXA scans were carried out in all study participants to assess percent fat mass (FM) and lean body mass (LBM) by using a QDR 4500 W–Explorer (Hologic Inc, Bedford, MA). Densitometric data analysis was performed by the software “Subregion Whole Body” which generates values in kilograms and percent of FMs and LBM for the whole body as well as for distinct body segments (upper limbs, trunk, lower limbs). The instrument radiation exposure during an examination of DXA was relatively low (1 mSv/d, micro-Sievert) according to the manufacturer.

During the insulin-free period LADA patients were treated with nutritional therapy, although in 58/210 patients (28%) oral hypoglycemic agents (OHA) were also added in order to improve the metabolic control. Insulin treatment was considered whenever patients displayed one of the following conditions: (i) early appearance of ketosis; (ii) significant body weight loss in spite of the usual diet; (iii) postprandial glucose level above 180 mg/dl despite maximum allowed dose of OHA.

Statistical analysis was performed using SPSS statistical software (version 16; SPSS, Chicago, IL). The values of anthropometric and DXA variables were expressed as means ± standard deviations. Differences between categorical variables were analysed by the Pearson χ^2^ test, whereas differences between scalar variables were tested using two-tailed Student’s *t* test for independent samples or ANOVA when more than two groups were compared. All comparisons were adjusted for age at recruitment and duration of disease. P-values ≤0.01 were considered statistically significant.

## Results

The clinical characteristics of all study participants are displayed in Table 1. Average BMI and waist circumference were significantly lower in LADA patients than in antibody-negative T2D (p < 0.0001) whereas they were significantly higher when compared to adult-onset T1D (p = 0.0002). The frequency of metabolic syndrome in the LADA group was 88/210 (42%), that is, intermediate between that of GADAb–negative T2D (87%) and adult-onset T1D (10%) and the differences between LADA and the other control groups were statistically significant (p < 0.001, chi–square).

**Table 1:** **Clinical characteristics of study participants**

Variables	LADA	GADAb-negative T2D	Adult-onset T1D	P-value
**Number of patients**	210	210	30	
**M:F**	92:118	92:118	11:19	n.s.^§^; n.s.^#^
**Age at onset (years)**	55.1 ± 10.6	57.7 ± 10.1	46.6 ± 7.9	n.s.^§^; <0.0001^#^
**Body mass index at onset (kg/m** ^**2**^ **)**	28.1 ± 5.3	30.8 ± 6.1	25.0 ± 3.5	<0.0001^§^; 0.0002^#^
**Waist circumference at onset (cm)**	95.2 ± 10.0	101.1 ± 12.1	86.3 ± 10.6	<0.0001^§^; 0.0002^#^
**Metabolic syndrome (%)**	88 (42%)	182 (87%)	3 (10%)	<0.0001^§^; 0.0008^#^

^§^LADA vs T2D; ^#^LADA vs adult–onset T1D.

### Whole body DXA

Mean values of body DXA parameters in the three groups of diabetes patients stratified by gender are reported in Table 2. In LADA patients the percent FM was similar to that of T1D and significantly lower compared to GADAb–negative T2D patients (P < 0.0001 in both genders). Percent LBM showed a similar trend, i.e. values in LADA patients were comparable to those of patients with adult-onset T1D and significantly higher than in GADAb-negative T2D patients (p = 0.0001). As expected, women showed on average 40-50% higher level of FM compared to men in all groups of patients.

**Table 2:** **Whole body composition in LADA, T2D and adult-onset T1D patients**

Variables	Gender	LADA	GADab-negative T2D	Adult-onset T1D	p-value
Fat mass (%)	M	21.6 ± 4.7	27.2 ± 5.9	21.3 ± 4.9	<0.0001^§^ ; n.s.^#^
	F	33.8 ± 5.5	37.1 ± 6.1	30.3 ± 6.8	0.0001^§^; n.s.^#^
Lean body mass (%)	M	75.2 ± 4.5	70.4 ± 6.5	75.6 ± 4.8	<0.0001^§^; n.s. ^#^
	F	63.5 ± 5.3	59.7 ± 6.9	66.8 ± 6.6	<0.0001^§^; n.s.^#^

^§^LADA vs T2D; ^#^LADA vs adult–onset T1D.

### Segmental DXA

Segmental DXA results are shown in Table 3. Among men, percent FM was significantly lower (p < 0.0001) in LADA than in T2D patients in all body segments (upper limbs, trunk and lower limbs), whereas no difference was detected between male LADA and adult-onset T1D patients. Among women, percent FM was significantly higher in LADA compared to antibody-negative T2D patients, but limited to the upper and lower segments, whereas in the truncal segment it was significantly lower (p < 0.0001). Compared to T1D women, LADA women displayed a significantly increased FM in the truncal segment, whereas no significant difference was found in the upper and lower compartments. Segmental LBM showed similar patterns in all groups but in reverse order.

**Table 3:** **Segmental body composition in LADA patients and control groups**

Variables	Gender	LADA	GADab-negative T2D	Adult-onset T1D	p-value
Upper limbs FM (%)	M	21.5 ± 5.3	29.7 ± 10.6	21.6 ± 5.8	<0.0001^§^ ; n.s.
	F	40.9 ± 6.9	31.2 ± 10.4	37.3 ± 11.8	<0.0001^§^ ; n.s.
Upper limbs LBM (%)	M	73.9 ± 5.3	66.4 ± 10.5	74.0 ± 5.6	<0.0001^§^ ; n.s.
	F	55.3 ± 6.8	64.9 ± 10.3	58.7 ± 11.3	<0.0001^§^ ; n.s.
Trunk FM (%)	M	21.0 ± 6.0	24.5 ± 6.5	20.0 ± 6.4	0.0002^§^ ; n.s.
	F	30.8 ± 8.0	38.1 ± 7.8	25.5 ± 6.8	<0.0001^§^ ; 0.005^#^.
Trunk LBM(%)	M	78.0 ± 6.0	74.8 ± 6.5	79.2 ± 6.4	0.0006^§^; n.s.
	F	68.5 ± 8.0	58.0 ± 7.8	73.8 ± 6.7	<0.0001^§^ ; 0.004^#^.
Lower limbs FM (%)	M	22.0 ± 4.4	27.0 ± 4.0	23.2 ± 4.8	<0.0001^§^; n.s.
	F	38.9 ± 5.3	28.5 ± 8.3	38.7 ± 9.2	<0.0001^§^ ; n.s.
Lower limbs LBM (%)	M	73.8 ± 4.3	69.5 ± 4.0	73.0 ± 4.8	<0.0001^§^; n.s.
	F	57.9 ± 5.2	67.6 ± 8.4	58.3 ± 9.0	<0.0001^§^; n.s.

^§^LADA vs T1D; ^#^LADA vs T2D.

### Progression of LADA patients towards insulin dependence according to BMI and BC parameters

The influence of BMI and BC parameters on the progression of diabetes was investigated by subdividing LADA patients into subgroups according to the median values of BMI, whole body FM and truncal FM. The proportion of LADA patients who became insulin-dependent after 4 years of follow up was 55% among those with a BMI below the median value (26 kg/m²) and only 30% among those with a BMI above this cut-off (p = 0.0001). The proportion of LADA patients with insulin dependence within 4 years was higher in patients with truncal fat below the median value (57% vs. 37%, p = 0.004), whereas no difference was observed when subgroups were defined according to the median value of the whole–body percent FM (52% vs. 43%, n.s.).

## Discussion

Latent autoimmune diabetes in adults accounts for roughly 10% of all cases of diabetes. The largest LADA cohorts analyzed so far [4–7] revealed a significant degree of heterogeneity of this form at the immunological level, whereas the existence of clinical heterogeneity is relatively less documented, especially for anthropometric and BC parameters. In this study we have analyzed the distribution of BMI and BC in a large cohort of LADA patients from the genetically homogeneous population of Sardinia [8], and compared the results with GADAb-negative T2D patients and T1D patients with onset after age 30.

Our data shows that the average BMI in the whole cohort of LADA patients is nearly 10% lower than in antibody-negative T2D patients and it displays a larger variability than in adult-onset T1D. This finding was further confirmed by BC analysis through DXA scans, which revealed that LADA patients as a whole have significantly less fat deposits than classical non-autoimmune T2D patients, substantially comparable to that of adult–onset T1D, with gender differences reflecting the physiological sexual dimorphism of adult subjects. Segmental BC analysis also revealed in LADA patients the existence of a peculiar distribution of fat into the body compartments, depending on gender. Among men, adipose tissue is significantly lower in LADA than in T2D patients with no appreciable differences across the three body segments. In contrast, in LADA women adipose tissue surpasses the proportion found in T2D in upper and lower body segments, whereas it is relatively reduced in the truncal segment. Thus, fat deposits in LADA women accumulate mostly in peripheral body segments, as observed in T1D women, although a subgroup of LADA women displays a truncal fat accumulation resembling that of T2D. A similar pattern in LBM distribution across the body compartments is observed as well, in parallel with the variations in FM. Taken together, these data indicate that in LADA women fat deposits tend to follow a more peripheral pattern that in classic, non-autoimmune T2D, whereas in men they follow a T1D-like pattern.

As for the relationship between fat deposits and disease progression, we found that a lower truncal fat percentage is a significant predictor of beta-cell exhaustion, which is consistent with our previous finding that LADA patients with low BMI values will develop a need for insulin treatment sooner than patients with higher BMI values [8]. We also confirm that the frequency of metabolic syndrome in LADA as a whole is significantly reduced compared to T2D, although higher than in adult-onset T1D patients.

The specific mechanisms accounting for the existence of distinct BC patterns in LADA subgroups are not known, and we can only speculate that they may be related to the severity of the underlying autoimmune process, which entails a variable duration of the residual beta cell function. As for the risk of late complications, it is known that excess abdominal fat increases the CV risk, whereas accumulation of fat in lower-body subcutaneous areas may be protective, as recently suggested by Lim et al. [10]. Since in T2D, especially women, adipose tissue deposits are larger in the truncal and upper segments and smaller in the lower body regions [11] this may explain, at least in part, why they show an increased risk of premature CV disease. On the same basis, we postulate that LADA women, showing specifically a peripheral fat accumulation may be more protected against CV risk compared with LADA women whose fat distribution is closer to that of T2D women. To what extent such patterns, if any, can influence the susceptibility to late complication is at present unknown, and deserves to be tested in cohorts with more extended follow–ups.

The present study has several strengths. First, the LADA study cohort belongs to a genetically homogeneous ethnic group, thus minimising the influence of a major confounder. Second, BC was determined through DXA which is considered the gold standard for assessing visceral and subcutaneous fat deposits, and it gives more accurate information than the simpler anthropometric measurements adopted in earlier studies. However, our findings are essentially descriptive and, at the moment, a consistent explanation of the BC heterogeneity found in LADA is not available, and the short follow–up period does not allow to test the impact of BC on the long-term outcome of the disease.

## Conclusions

In a cohort of LADA patients, we observed a BC different from that of non–autoimmune T2D, the former being much closer to adult–onset T1D, as pointed out by previous studies. The relative depletion of visceral fat observed in LADA patients is associated with a faster progression towards insulin dependence. We suggest that the LADA patients with larger fat mass in the peripheral regions may be exposed to a lower risk of late cardiovascular complications than in T2D, a hypothesis that deserves further testing in larger clinical trials.
